# Blends of Organic Acids Are Weaponizing the Host iNOS and Nitric Oxide to Reduce Infection of *Piscirickettsia salmonis* in vitro

**DOI:** 10.3390/antiox13050542

**Published:** 2024-04-28

**Authors:** Nicolae Corcionivoschi, Igori Balta, David McCleery, Ioan Pet, Tiberiu Iancu, Calin Julean, Adela Marcu, Lavinia Stef, Sorin Morariu

**Affiliations:** 1 Bacteriology Branch, Veterinary Sciences Division, Agri-Food and Biosciences Institute, Belfast BT4 3SD, Northern Ireland, UK; david.mccleery@afbini.gov.uk; 2 Faculty of Bioengineering of Animal Resources, University of Life Sciences King Mihai I from Timisoara, 300645 Timisoara, Romania; ioanpet@usvt.ro (I.P.); calinjulean@usvt.ro (C.J.); adelamarcu@usvt.ro (A.M.); laviniastef@usvt.ro (L.S.); 3 Academy of Romanian Scientists, Ilfov Street, No. 3, 050044 Bucharest, Romania; 4 Faculty of Management and Rural Development, University of Life Sciences King Mihai I from Timisoara, 300645 Timisoara, Romania; 5 Faculty of Veterinary Medicine, University of Life Sciences King Mihai I from Timisoara, 300645 Timisoara, Romania; sorin.morariu@fmvt.ro

**Keywords:** *Piscirickettsia salmonis*, natural antimicrobials, antimicrobial peptides, iNOS, NO, inflammation

## Abstract

For the last 30 years, *Piscirickettsia salmonis* has caused major economic losses to the aquaculture industry as the aetiological agent for the piscirickettsiosis disease. Replacing the current interventions, based on antibiotics, with natural alternatives (e.g., organic acids) represents a priority. With this study, we aimed to better understand their biological mechanism of action in an in vitro model of infection with salmon epithelial cells (CHSE-214). Our first observation revealed that at the sub-inhibitory concentration of 0.5%, the organic acid blend (Aq) protected epithelial cell integrity and significantly reduced *P. salmonis* invasion. The MIC was established at 1% Aq and the MBC at 2% against *P. salmonis*. The sub-inhibitory concentration significantly increased the expression of the antimicrobial peptides Cath2 and Hepcidin1, and stimulated the activity of the innate immune effector iNOS. The increase in iNOS activity also led to higher levels of nitric oxide (NO) being released in the extracellular space. The exposure of *P. salmonis* to the endogenous NO caused an increase in bacterial lipid peroxidation levels, a damaging effect which can ultimately reduce the pathogen’s ability to attach or multiply intracellularly. We also demonstrate that the increased NO release by the host CHSE-214 cells is a consequence of direct exposure to Aq and is not dependent on *P. salmonis* infection. Additionally, the presence of Aq during *P. salmonis* infection of CHSE-214 cells significantly mitigated the expression of the pro-inflammatory cytokines IL-1β, IL-8, IL-12, and IFNγ. Taken together, these results indicate that, unlike antibiotics, natural antimicrobials can weaponize the iNOS pathway and secreted nitric oxide to reduce infection and inflammation in a *Piscirickettsia salmonis* in vitro model of infection.

## 1. Introduction

Salmonid Rickettsial Septicaemia (SRS), or Piscirickettsiosis, is the principal infectious disease in salmon aquaculture [1]. Triggered by the pathogen *Piscirickettsia salmonis*, this disease is responsible for annual economic damages conservatively estimated at around 700 million dollars [2,3]. Discovered in 1989 within farmed coho salmon (*Oncorhynchus kisutch*) in Chile, *Piscirickettsia salmonis*, a Gram-negative facultative intracellular bacterium, resembles a rickettsia-like organism identified as a pathogen in fish [4,5]. Phylogenetically, the pathogen shows closer similarities to species within the *Legionella*, *Francisella*, and *Coxiella* genera than members of the *Rickettsia* genus [6]. Since its initial identification, *P. salmonis* has been recognized as the pathogenic agent responsible for both clinical and chronic forms of Salmonid Rickettsial Septicaemia (SRS) in coho salmon (Oncorhynchus kisutch), Atlantic salmon (*Salmo salar*), rainbow trout (*Oncorhynchus mykiss*), white seabass (*Atractoscion nobilis*), European seabass (*Dicentrarchus labrax*), and groupers (*Epinephelus melanostigma*) across various countries, including Norway, Canada, Scotland, and Ireland [3,7]. Despite the availability of 33 commercial vaccines against piscirickettsiosis and the use of antibiotics as primary control measures, the effectiveness of these strategies under real-world conditions remains suboptimal, highlighting the challenges in managing this disease effectively and the necessity of novel antimicrobial strategies [4,8]. The pathogenesis of SRS in salmonids is commonly associated with *P. salmonis* isolates resembling the LF-89 and EM-90 strains, which have distinct infectivity characteristics [3,9]. Furthermore, the planktonic EM-90 isolate and the sessile LF-89 demonstrated the highest virulence, linked to the differential expression of IL-1β, IL-8, NF-κB, and IKB-α genes in SHK-1 cells [9]. For example, fish infected with the EM-90 strain exhibit more increased cumulative mortality and a shorter time to death compared to those infected with LF-89 [3]. Infections caused by LF-89 typically present a more classic SRS pathology, including splenomegaly, renomegaly, prominent yellowish-white nodules in the kidney and liver, fibrinous pseudo membranes in the liver and heart, hydropericardium, and ecchymosis in the liver. Conversely, the EM-90 isolate leads to an acute systemic and haemorrhagic condition, with lesions affecting all internal organs [3].

Mixtures of organic acids can protect epithelial cells from bacterial-induced damages, indicating their beneficial effects and the potential to be considered a substitute to antibiotic treatment [10]. Employing natural antimicrobial strategies in aquaculture could enhance production sustainability and decrease antibiotic reliance. These approaches appeal because of their simple preparation processes, cost-effectiveness, reduced environmental impact, and scientific support for using combinations of organic acids with plant extracts, essential oils, phytochemicals, and probiotics. Their antimicrobial, antioxidant, and antiviral properties, alongside their capacity to support the immune system depicted in in vivo and in vitro studies, have contributed to improved fish health and welfare [5]. In addition, phytochemicals are recognized for their role in preventing viral and bacterial adhesion to cells and influencing intracellular mechanisms [5]. These actions stimulate innate and adaptive immune responses, encompassing cellular and humoral immunity. Mechanistically, these protective effects observed in these studies were correlated to the early activation of INF-II and effector proteins like the C3 complement factor and CD8+ T cells, creating a protective microenvironment versus *P. salmonis* [5]. Other mechanisms might trigger the NF-kβ pathway and initiation of a pro-inflammatory transcriptional response [11]. The intracellular behaviour of *P. salmonis* and its sophisticated infection tactics clarify why this pathogen effectively causes disease and why current antibiotic treatments often fail. Hence, there is a pressing need to pave the way to new non-pharmacological approaches to combat this bacterium, intending to apply them commercially.

Recently, natural antimicrobials were proven to have an enhanced effect against various viral [12] and bacterial pathogens [13] when used as blends. With this study, we aimed to better understand the biological mechanisms of inhibition in *Piscirickettsia salmonis* by using in vitro models which could provide the vital initial steps for further in vivo trials given the devastating effect of these infections in salmon. This will not only provide vital data for advanced experimental design but could significantly improve our understanding of how these alternatives to antibiotics express their antipathogenic effect rather than bactericidal.

## 2. Materials and Methods

### 2.1. Piscirickettsia salmonis, CHSE-214 Cell Lines and Organic Acid Blend

The CHSE-214 epithelial cells were obtained from the European Collection of Authenticated Cell Cultures (ECACC). The CHSE-214 cells (ECACC No. 91041114) are a fish cell line derived from *Oncorhyncus tshawytscha* embryos and previously used in shrimp-related studies [14]. They were cultured in minimum essential medium (MEM) (Gibco) supplemented with 10% foetal bovine serum (Life Technologies), 2 nM L-glutamine (Corning), and an antibiotic/antimycotic solution (10,000 IU/mL penicillin, 10,000 μg/mL streptomycin and 25μg/mL amphotericin B). *Piscirickettsia salmonis* (*P. salmonis*) ATCC-VR-1361 was grown as previously described to produce bacterial pellets for infection and for growth curves [15]. The organic acid mixture (AuraAqua—Aq) contains lactic acid, E330 citric acid and citrus extract, 5% maltodextrin, 1% sodium chloride, 42% citric acid, 18% sodium citrate, 10% silica, 12% malic acid, 9% citrus extract, and 3% olive extract (*w*/*w*). The raw materials were supplied by Bioscience Nutrition Ireland.

### 2.2. Minimum Inhibitory (MIC) and Minimum Bactericidal (MBC) Concentration against P. salmonis

Both the MIC and the MBC were determined as previously described [16]. The two-fold tube dilution method was used to determine the lowest concentration of the inhibited bacterial growth (MIC) and the lowest concentration that induced bacterial death (MBC). AuraAqua was diluted (8% to 0.015625% *v*/*v*) in BM4 broth and vortexed. Individual overnight bacterial cultures were harvested by centrifugation, washed twice in PBS, and resuspended in BM4 media to 1 × 10^6^ CFU/mL. Each tube was inoculated with 5 × 10^5^ CFU/mL of each bacterial culture (final concentration). Non-inoculated bijou (5 mL) tubes containing the same growth medium were used as negative controls, whilst MHB tubes without AuraAqua were inoculated with individual bacterial cultures as positive controls. The *P. salmonis* tubes were incubated at 37 °C for 48 h. Tubes that did not show visible growth were above the MIC. One hundred millilitres were taken from each tube for inoculation and then incubated at 37 °C for 24 h onto BM4 agar at 37 °C for 48 h. The highest dilution of each antimicrobial with no microbial growth was considered as the MBC. The antimicrobial mixture was tested using concentrations from 8 to 0.0078% (*v*/*v*) in three independent replicates repeated three times for each strain. To determine the sub-inhibitory concentrations used, all three pathogens were exposed to different concentrations of the antimicrobial mixture. The highest concentrations of antimicrobial that showed no effect on survivability and no growth inhibition (same growth kinetics as the control) were used in subsequent experiments.

### 2.3. In Vitro Infection Assay and Intracellular Bacterial Quantification

The infection assay was performed as previously described [14]. Briefly, monolayers of CHSE-214 cells were prepared in 24-well plates at 1 × 10^6^ cells/well. Infection of CHSE-214 was performed as previously described with small modifications [17]. For infection, *P. salmonis* was grown in BM4 media at 23 °C to an OD_600_ of 0.3. A volume of 200 µL of bacterial culture was used to infect the CHSE-214 cells, cells were infected for another 5 and 10 days of infection. The total intracellular bacteria were enumerated at 5 and 10 days post infection. The experimental design included bacteria pre-treated for 30 min at 23 °C with 0.5% Aq in DMEM. Secondly, the CHSE-214 cells were pre-treated with 0.5% Aq for 1 h prior to infection, and thirdly, a concentration of 0.5% Aq was used during each infection experiment without prior treatment of bacteria or the CHSE-214 cells. CHSE-214 cells (80% confluent) were infected with stationary phase bacteria at a multiplicity of infection (MOI) of 100 at 23 °C. At 5 or 10 days post infection, the infected monolayers were washed with ice cold 0.1% Saponin in PBS (3×) for 15 min at 16 °C to permeabilize the cells. Cells were diluted in PBS (1×) collected by centrifugation (6000× *g*) for 10 min at 4 °C. Quantification of intracellular bacteria was performed by qPCR as previously described [18] using the primers described in Table 1.

### 2.4. Quantification of Antimicrobial and Anti-Inflammatory Activity in CHSE-214 Cells Infected with P. salmonis in the Presence of 0.5% Aq

The expression of Cath2, Hepcidin1, iNOS, IFNγ, IL-8, IL-12, and IL-1β was measured in CHSE-214 cells infected with bacteria pre-treated for 30 min at 23 °C with 0.5% AuraAqua in DMEM. The expression was also quantified in CHSE-214 cells pre-treated with 0.5% AuraAqua for 1 h prior to infection and in cells infected in the presence of a concentration of 0.5% AuraAqua. The CHSE-124 cells were snap-frozen in liquid nitrogen until use. RNA was isolated using the RNeasy Plus Mini Kit (Qiagen, Manchester, UK). The RNA was reverse transcribed using the Transcriptor First Strand cDNA Synthesis Kit (Roche) according to the manufacturer’s protocol. The mRNA levels were determined by quantitative RT-PCR using the QuantiNovaSYBR Green PCR Kit (Qiagen, Manchester, UK) on a LightCycler 96 (Roche). The qPCR reaction included 1 μL of cDNA template in a final volume of 10 μL. Water was used as control. The real-time PCR was performed in 96-well PCR plates at 50 °C for 2 min, followed by a 95 °C hot start for 10 min; the amplification was performed by 40 cycles of 95 °C for 15 seconds (sec) and 60 °C for 1 min. qPCR primers were used in a final concentration of 0.25 μM. The primers used are included in Table 1. The 2^−ΔΔCT^ method was used to analyse the relative expression (fold changes), calculated relative to the control group. To estimate the gene expression, untreated cells (control) were set as value 1 and treated cells’ values were compared to this value. Each experiment was repeated 3 times and on 3 separate occasions. The stimulated sample was compared to its control at each time point. *p*-values ≤ 0.05 were considered significant.

### 2.5. Determination of Aq Cytotoxicity and and LDH Release

To measure any antimicrobial effect on epithelial cell proliferation, the cells were cultured as described above and treated with a series of concentrations of AuraAqua (0, 0.5, 1, 2%) for 5-10 days to examine the dose and time-dependent AuraAqua impact on CHSE-214 cell viability using an MTT (3-(4,5-dimethylthiazol-2-yl)-2,5-diphenyl-tetrazolium bromides) assay at 37 °C. The results were shown in a graph of the percentage of cell viability (% cell viability = [A_treated cells_/A_untreated cells_] × 100) against concentrations. The CHSE-214 cells were grown on 24-well plates (Thermo Fischer Scientific) at 1 × 10^5^ cells/well as described above. Infections were performed with *P. salmonis* grown in BM4 broth at a concentration of MOI 100. Cells were infected for another 5 and 10 days of infection. Media were removed from each well to measure lactate dehydrogenase (LDH)-based cytotoxicity. Lactate dehydrogenase release (LDH) was measured with a cytotoxicity detection kit (Roche, Buckinghamshire, UK) following the manufacturer’s instructions by a spectrophotometric reading at 500 nm (FLUOstar Omega, Premier Scientific, UK). The results are expressed as cytotoxicity calculated as a percentage of the total lysis of cells lysed in Triton ×100.

### 2.6. Nitric Oxide (NO) Measurement in Infected CHSE-214 Cells Treated with Aq

Nitrate concentrations in infected cells were measured as previously described using the nitric oxide assay kit (ab233469, Abcam, UK) [21]. Measurements were performed in cells infected with bacteria pre-treated for 30 min at 23 °C with 0.5% Aq in DMEM. Nitric oxide was also measured in CHSE-214 cells pre-treated with 0.5% Aq for 1 h prior to infection and in infected CHSE-214 cells in the presence of 0.5% Aq. Controls also included NO measured in CHSE-214 cells only, and 10 mM 4-diamino-6-hydroxypyrimidine (DAHP) [22] as an iNOS inhibitor (Thermo Fischer Scientific, UK) and 50 μg/mL *E. coli* LPS (Thermo Fischer Scientific, UK) [23]. The absorbance was measured spectrophotometrically at 540 nm (FLUOstar Omega, Premier Scientific, UK) and concentrations calculated from the standard curve following the manufacturer’s instructions. All experiments were performed in triplicate.

### 2.7. P. salmonis Lipid Peroxidation (TBARS Assay)

The TBARS assay was conducted as previously described [24]. The *P. salmonis* lipid peroxidation assay was performed in 0.2 μM transwell filter chambers to ensure no physical contact between the bacteria and the CHSE-214 cells and to allow us to account for the NO release as a consequence of 0.5% Aq exposure only. Measurements were performed 10 days post infection and the experimental design included: (A) CHSE-214 cells only; (B) *P. salmonis*-infected cells (10^9^ CFU/mL); (C) CHSE-214 + 50 μg/mL LPS; (D) CHSE-214 + 50 μg/mL LPS + *P. salmonis*; (E) CHSE-214 + 10mM DAHP + *P. salmonis* + 0.5% Aq; and (F) CHSE-214 + *P. salmonis* + 0.5% Aq. The NO levels were measured in all experiments as described above. For lipid peroxidation, bacteria from experiments B, D-F, were collected and TBARS were determined by using a commercial kit (Abcam, ab118970, UK). The bacteria were suspended in 50 μL of sodium dodecyl sulphate with 1.25 mL thiobarbituric acid buffer and incubated at 95 °C for 60 min. After incubation and centrifugation at 5000× *g* for 15 min, fluorescence of the supernatant was measured at an excitation of 530 nm and emission at 550 nm using a spectrofluorometer (FLUOstar Omega, Premier Scientific, UK). The fluorescence intensity was converted to malondialdehyde (MDA) equivalent per 1010 cells using an MDA standard curve. All experiments were performed in triplicate.

### 2.8. Statistical Analysis

Statistical analyses were performed using GraphPad software, version 10 (Boston, MA, USA). Data were represented as mean ± SD. Significance was assigned at *p*-values < 0.05 following estimations using the two-tailed Student’s *t*-test.

## 3. Results

### 3.1. Minimum Inhibitory Concentration (MIC), Minimum Bactericidal Concentration (MBC), and the In Vitro Cytotoxic Effect of Aq

**Figure 1:**
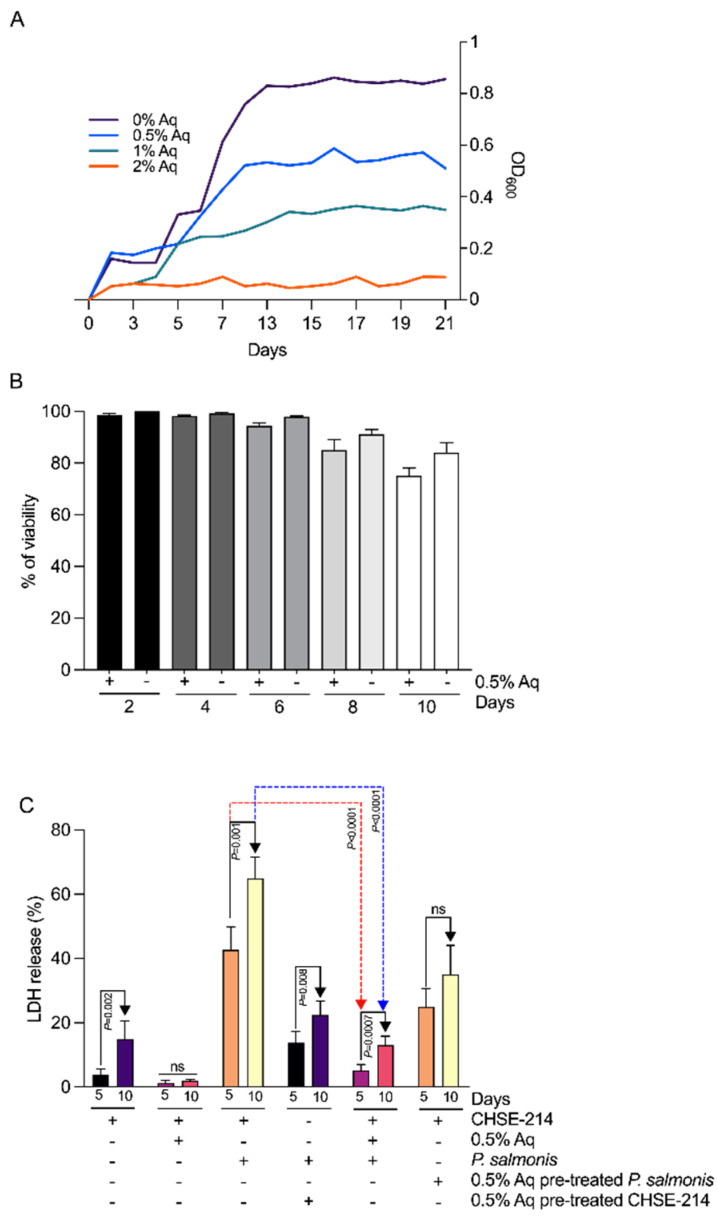
The effect of the organic acid mixture (Aq) on in vitro cell invasiveness by *P. salmonis* using CHSE-214 cells. (Panel **A**) shows the MIC and the MBC concentrations at which Aq influences the growth of P. salmonis. (Panel **B**) shows CHSE-214 cell viability as determined by the MTT assay and expressed as a percentage of the untreated control cells. (Panel **C**) shows the cytotoxic effect of Aq to CHSE-214 cells as determined following 4 and 10 days by LDH release. The experiments were per formed in triplicate and on three separate occasions. The *p*-values following a Student’s *t*-test analysis, to assess the impact of Aq, are presented on the graph.

First, the MIC was established at 1% Aq and the MBC at 2% Aq (Figure 1A). As indicated in Figure 1A, the concentration of 0.5% Aq was sub-inhibitory and was chosen to further investigate the biological mechanisms involved in its antibacterial effect. At this concentration, there was no significant effect on CHSE-214 cell viability (Figure 1B). To further investigate the potential cytotoxic effect, we measured the levels of lactate dehydrogenase (LDH) release from infected CHSE-214 cells. As indicated in Figure 1C, the levels of LDH release were incremental from 5 to 10 days in the *P. salmonis*-infected CHSE-214 cells, and significantly decreased following the inclusion of 0.5% Aq during the infection assay (*p* < 0.0001). The lowest decrease in LDH release was detected when pre-treated *P. salmonis* bacteria were used to infect unexposed CHSE-214 cells. These results clearly suggest that at the sub-inhibitory concentration of 0.5%, Aq protects the cellular integrity, potentially by preventing *P. salmonis* invasion.

### 3.2. In Vitro, the Organic Acid Blend Prevents P. salmonis Infection in CHSE-214 Cells

**Figure 2:**
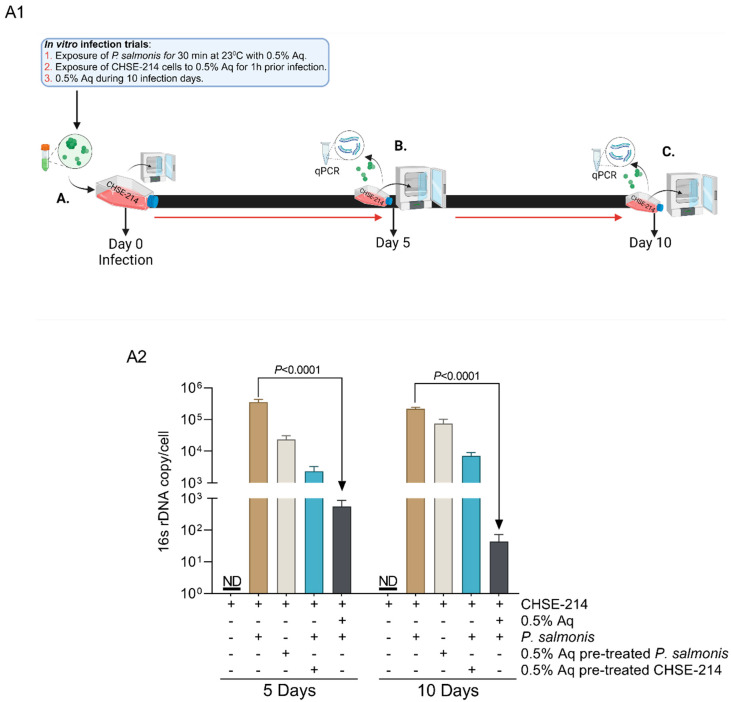
In vitro effect of Aq on CHSE-214 cell invasiveness by *P. salmonis*. (**Panel A1**)—in vitro infection assay design. Designed with Biorender.com. Cells were infected at day 0 (MOI 100) (**Panel A1-A**) and quantified at days 5 and 10 post-infection (**Panel A1-B**) to (**A1-C**). (**Panel A2**)—quantification of infection levels by qPCR at 5 and 10 days post infection. The experiments were performed in triplicate and on three separate occasions and *p*-values following a Student’s *t*-test analysis to assess the impact of Aq are presented on the graph.

To further prove that the decrease in LDH release, in the presence of 0.5% Aq, appears because of lower infection levels, we next performed three in vitro infection assays as described in Figure 2(A1). The infection was measured at 5 and 10 days post infection. This approach allowed us to estimate the impact of Aq on the *P. salmonis* intracellular numbers post infection. The results presented in Figure 2(A2) show that at 5 days post infection, the numbers of bacteria internalised were significantly decreased in the presence of 0.5% Aq (*p* < 0.0001). We also show that at 10 days post infection, *P. salmonis* was unable to further multiply once internalised in the CHSE-214 cells, suggesting structural bacterial damage. These data suggest that Aq could increase the antibacterial host defence mechanisms and eradicate internalised bacteria.

### 3.3. The Effect of Aq on Immune Gene Expression in P. salmonis-Infected CHSE-214 Cells

**Figure 3:**
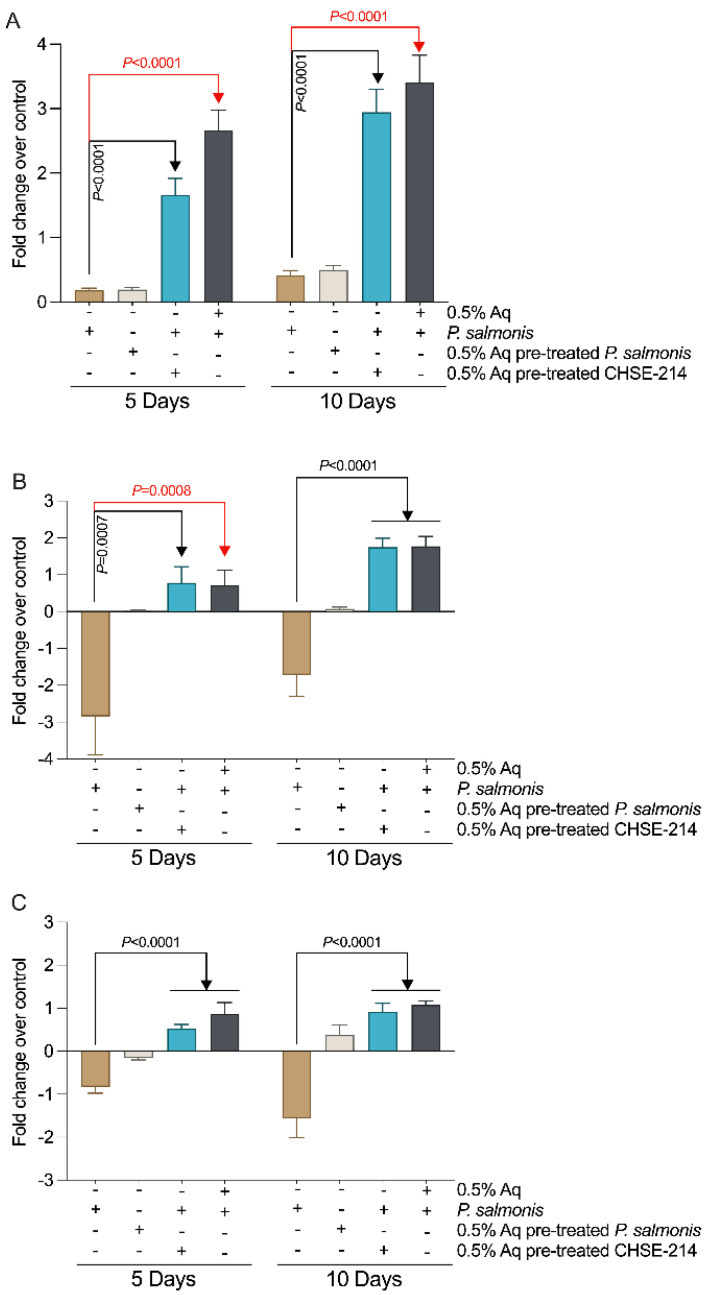
The impact of Aq on gene expression in CHSE-214 cells during infection with *P. salmonis*. The cells were infected as described in Figure 2A. Gene expression for Cath2 (**A**), Hepcidin-1 (**B**), iNOS (**C**) was quantified using qPCR and compared to the controls at 5 and 10 days post infection. Fold differences were calculated in respect to the mean of the controls at the three time points. The experiments were performed in triplicate and on three separate occasions and *p*-values following a Student’s *t*-test analysis to assess the impact of Aq are presented on the graph.

Next, we aimed to investigate if the stimulated host antibacterial mechanisms could include peptides of enzyme pathways, which might help the host to fight against bacterial invasion. We did so by investigating if 0.5% Aq has an impact on the innate immune response in *P. salmonis*-infected CHSE-214 cells. To achieve this, we measured the expression of antimicrobial peptide production genes, cathelicidin (Cath2), hepcidins (Hepcidin-1), and the innate immune effector iNOS. Our results show that the inclusion of 0.5% Aq during infection or the pre-treatment of the CHSE-214 cells with 0.5 Aq has a significant (*p* < 0.0001) effect on Cath2 (Figure 3A) at both 5 and 10 days post infection. The expression of Hepcidin 1 (Figure 3B) was also significantly stimulated by Aq at both 5 days (*p* = 0.0007 and *p* = 0.0008) and 10 days post infection (*p* < 0.0001). A similar effect was also noticed when the expression of iNOS was monitored (*p* < 0.0001). No significant effect was observed on the expression of these peptide production genes when *P. salmonis* was pre-treated with 0.5% Aq before infection of the CHSE-214 cells. These results suggest that Aq has indeed a positive impact in stimulating the host immune system to produce antimicrobial peptides and fight the *P. salmonis* invasion of CHSE-214 cells.

### 3.4. Nitric Oxide (NO) Levels in P. salmonis-Infected CHSE-214 Cells and the Impact of Aq

**Figure 4:**
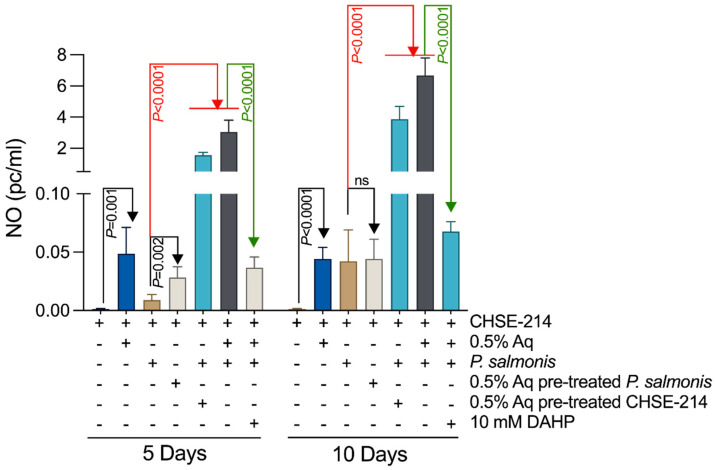
The effect of Aq on the NO levels produced in *P. salmonis*-infected CHSE-214 cells. The experiments were performed in triplicate and on three separate occasions and *p*-values following a Student’s *t*-test analysis to assess the impact of Aq are presented on the graph.

The stimulation of iNOS expression during infection of *P. salmonis*, in the presence of 0.5% Aq, and the increased NO release, led us to believe that nitric oxide (NO) might be potentially responsible for the reduced bacterial invasion. In Figure 4, our experimental data show that at 5 days post infection, 0.5% Aq had a significant impact (*p* < 0.0001) on NO release when the cells were treated or when Aq was constantly present during the infection assay. Similar effects were detected when NO measurements were performed 10 days post infection (*p* < 0.0001). The addition of 10 mM DAHP inhibitor to the infected CHSE-214 cells significantly reduced NO release in the presence of 0.5% Aq (*p* < 0.0001). The observed increase in NO release upon infection, in the presence of Aq, suggests that nitric oxide can act as an antimicrobial molecule and reduce *P. salmonis* infection of CHSE-214 cells. This antimicrobial effect can include regulation of the host’s pro-inflammatory response.

### 3.5. The Effect of 0.5% Aq on the Pro-Inflammatory Cytokine Levels’ Expression in P. salmonis-Infected CHSE-214 Cells

**Figure 5:**
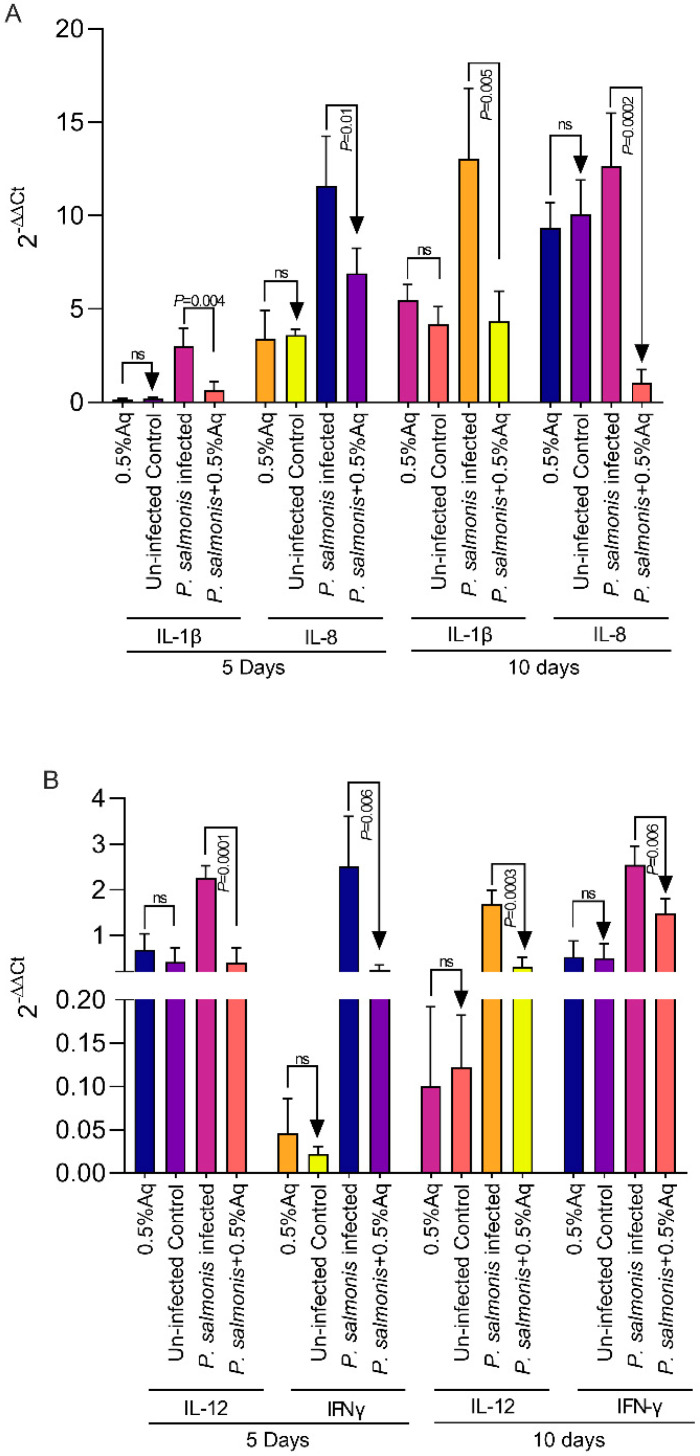
The impact of Aq on pro-inflammatory gene expression in CHSE-214 cells during infection with *P. salmonis*. The cells were infected as described for 5 and 10 days in the presence/absence of 0.5% Aq. Cells in the absence of 0.5% Aq only and uninfected cells were used as controls. Gene expression for IL-1β and IL-8 (**Panel A**), and IL-12 and IFNγ (**Panel B**) was measured by qPCR and compared to the controls at 5 and 10 days post infection. Data are expressed as 2^−ΔΔCt^ where ΔCt  =  Ct (target gene) − Ct (housekeeping); values are the mean of three test replicates. The experiments were performed in triplicate and on three separate occasions and *p*-values following a Student’s *t*-test analysis to assess the impact of Aq are presented on the graph.

To test the hypothesis that the Aq-induced NO production can be involved in *P. salmonis* pathogenesis in CHSE-214 cells, we measured the expression of IL-1β, IL-8, IL-12, and IFNγ cytokines. Our results show a significant decrease in IL-1β (*p* = 0.004), IL-8 (*p* = 0.01), IL-12 (*p* < 0.0001), and IFNγ (*p* = 0.006) (Figure 5A,B) at 5 days post infection in the presence of 0.5% Aq. At 10 days post infection, the expression of IL-1β (*p* = 0.005), IL-8 (*p* = 0.0002), IL-12 (*p* = 0.0003), and IFNγ (*p* = 0.006) was also significantly affected (Figure 5A,B). These results clearly show that the presence of Aq during the infection of *P. salmonis* of CHSE-214 cells significantly attenuates the expression of the IL-1β, IL-8, IL-12, and IFNγ pro-inflammatory cytokines, a reduction which takes place alongside an increase in iNOS expression and NO release by the infected cells.

### 3.6. The Impact of CHSE-214-Released Nitric Oxide on P. salmonis

**Figure 6:**
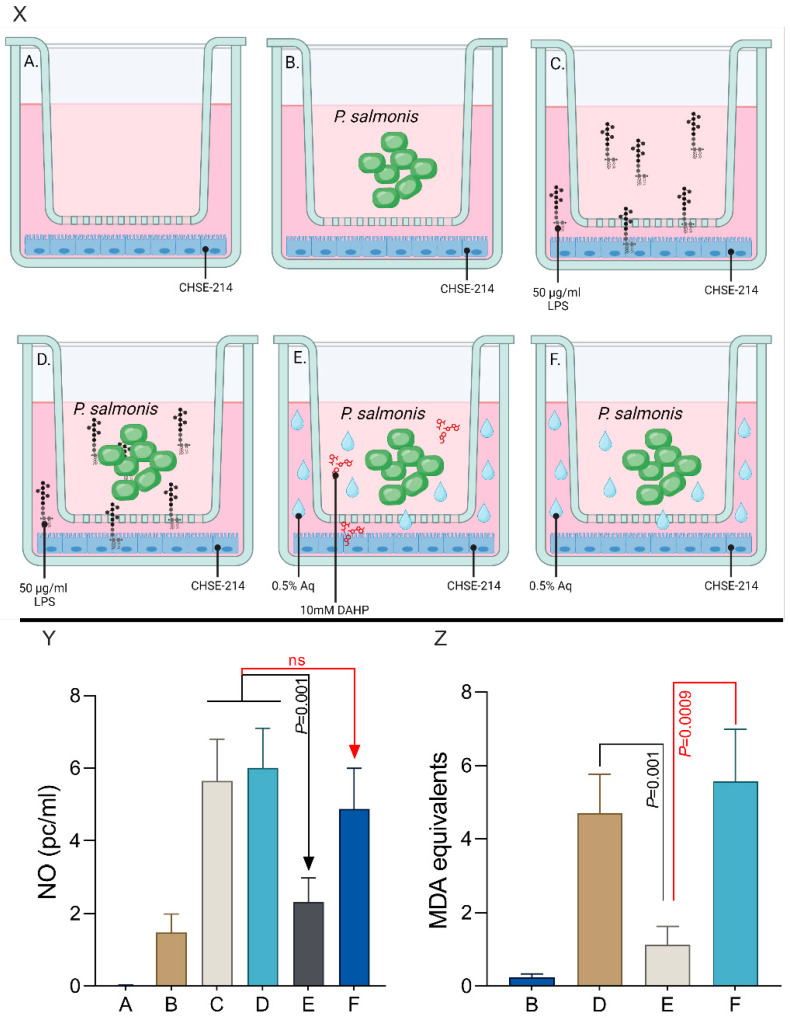
The levels of extracellular NO release and bacterial lipid peroxidation levels during coculture in the presence of 0.5% Aq. (**Panel X**)—experimental design. Designed with Biorender.com. (**Panel Y**)—NO levels in the coculture supernatant. (**Panel Z**)—MDA equivalents to express levels of bacterial lipid peroxidation. The experiments were performed in triplicate and on three separate occasions. The *p*-values following a Student’s *t*-test analysis, to assess the impact of Aq, are presented on the graph.

Next, we aimed to identify if the NO, released by the CHSE-214 cells, upon exposure to 0.5% Aq, has a direct impact on *P. salmonis*. There was no physical contact between *P. salmonis* and the epithelial cells. Our experimental design aimed to: (1) identify if Aq is responsible for triggering NO release by the CHSE-214 cells and (2) to investigate the effect of cell-produced NO on the *P. salmonis* bacterial integrity. The experimental design is described in the Materials and Methods Section and is graphically shown in Figure 6X (Panels A–F). The NO levels were not significant in the cells-only media (Figure 6(YA)) or when *P. salmonis* was added in the chambers (Figure 6(YB)). CHSE-214 cells’ stimulation with LPS led to a significant increase in NO release (Figure 6(YC)). When LPS was added to the chamber, in the presence of *P. salmonis* (Figure 6(YD)), the levels of NO were like those detected in Figure 6(YC), suggesting that *P. salmonis* is not involved in triggering iNOS. When iNOS activity was inhibited with DAHP (Figure 6(YE)), a significantly lower NO secretion was detected (*p* = 0.001). When DAHP was removed (Figure 6(YF)), the levels of NO mimicked LPS stimulation. To assess the impact on bacteria, *P. salmonis* cells were harvested and levels of lipid peroxidation were measured as described in the Materials and Methods Section (Figure 6(XB), D–F). As indicated in Figure 6(ZB), the levels of lipid peroxidation in *P. salmonis* exposed to CHSE-214 cells only were almost at undetectable levels. When LPS is added and the NO levels are elevated (Figure 6(YD)), a significant increase (*p* = 0.001) in lipid peroxidation is detected (Figure 6(ZD)) compared to the levels detected in *P. salmonis* isolated from the chamber including the iNOS inhibitor (Figure 6(ZD)). When 0.5% Aq was added in the chamber (Figure 6(ZF)), the levels of lipid peroxidation in the exposed *P. salmonis* were similarly significantly increased (*p* = 0.0009). No *P. salmonis* was detected in the transwells’ lower chamber as probed by PCR to control for filter efficacy (data not shown). These results suggest that Aq can stimulate iNOS expression and subsequent NO release in the extracellular space followed by induced *P. salmonis* structural damages and a reduced ability to attach or multiply intracellularly.

## 4. Discussion

*P. salmonis* can endure and proliferate within host macrophages, monocytes, and non-lymphoid cells, effectively circumventing the immune response [3,5]. In vivo and in vitro findings indicated that *P. salmonis* persisted for ≥120 h or more in macrophage-enriched cell cultures derived from Atlantic salmon, with a concurrent peak in identifying the 16S rDNA copy number per cell [18]. It infects, resides, and multiplies primarily within the cytoplasmic vacuoles of macrophages and polymorphonuclear leukocytes without triggering a notable cytopathic effect [3]. In vitro studies have shown that *P. salmonis* replication is most efficient at temperatures between 15 and 18 °C, with a notable decline in the replication rate at 21 °C [8]. *P. salmonis* can persist for up to 14 days in salt water, yet it is nearly instantaneously neutralized upon exposure to freshwater [8]. *P. salmonis* can attach to living surfaces and form biofilms on non-living (abiotic) substrates such as plastic, glass, and mussel shell [8,25,26,27]. This capacity for biofilm formation is facilitated by the expression of the *che*A and *psl*D chemotaxis genes, which play a vital role in promoting the development of biofilms. This is linked to the development of what is referred to as the “piscirickettsial attachment complex” [25,26]. This mechanism facilitates the bacterium’s adherence to various surfaces and may enhance the vertical transmission of piscirickettsiosis, particularly when *P. salmonis* binds to fish eggs [25,27]. The ability of *P. salmonis* to attach to and infect CHSE-214 cells in the presence of the antimicrobial mixture (Aq) used in this study was significantly reduced, suggesting its potential in mediating a host’s defence mechanism to reduce its invasion capabilities. The anti-infective effect of Aq extends to the pathogen itself, since pre-exposure of *P. salmonis* to Aq also reduced its ability to infect CHSE-214 cells; however, further investigations are necessary to uncover its effects.

Increased concentrations of nitric oxide (NO) lead to its potential binding to DNA molecules, proteins, and lipids, and ultimately result in pathogen killing [28]. Previously, we have shown that Aq was able to stimulate the growth of the probiotic bacterium *Faecalibacterium prausnitzii* in shrimp and prevent *Vibrio parahaemolyticus* infections [29]. This antipathogenic mechanism was described for the probiotic bacterium *Lactobacillus rhamnosus* GG, which can induce NO production in epithelial cells by activating iNOS activity via the NF-κB pathway [30]. The role of Aq in modulating the activity of NF-κB was previously described in shrimp primary epithelial cells, a study which suggested that Aq has an involvement in reducing H_2_O_2_ production by the infected cells but could not explain why the catalase and superoxide dismutase were elevated [31]. One explanation could be that catalase [32] and superoxide dismutase [33] were shown to regulate iNOS expression in Raw cells. In this study, upon iNOS activation, the presence of Aq during infection leads to an incremental release of NO by the CHSE-214 cells and to a decrease in *P. salmonis* infection. The release of NO in solution contributes to bacterial eradication [34] through oxidative damage inflicted on bacterial structures (e.g., lipid peroxidation or nitrosation of membrane proteins) [35]. We designed our infection experiments to ensure that bacterial phase variation is also taken in account since in the case of other intracellular pathogens, iNOS activation is dependent on the alteration of bacterial surface structures [36]. We believe that *P. salmonis* was not subjected to phase variations that could protect the bacteria against the destructive effect of the released NO since we discovered that intracellular proliferation was not possible in the presence of Aq; hence, the decrease in the invasion levels.

Additionally, our data show that when present during infection, Aq is responsible for the increase in NO release extracellularly. This was proven in a transwells experiment which prevented physical contact between the bacteria and the CHSE-214 cells. The secreted NO, following stimulation of iNOS, was able to induce a significant level of lipid peroxidation in planktonic *P. salmonis*, suggesting a plausible explanation for the observed decreased levels of invasion. Moreover, NO can protect tight junctions against reactive oxygen species-induced damage since there is evidence that hydrogen peroxide-induced tight junction protein dephosphorylation and barrier disruption was mitigated by the inclusion of an NO donor [37].

*P. salmonis* activates phagocytosis by interacting with the host cell’s clathrin and actin [38]. The process is further characterized by the phosphorylation-dependent activation of the IKB kinase (IKK) complex, leading to the subsequent nuclear translocation and activation of NF-κB. NF-κB, in turn, binds to specific promoters to initiate the expression of inflammatory cytokines, including TNF-α, IL-6, IL-8, IL-1β, IL-18, IL-10, and IFN-γ [38]. This phenomenon has been previously observed in several fish cell lines, such as SHK-1 and RTS11, as well as in Atlantic salmon [38]. Our data indicate that the antimicrobial mixture used in this study significantly downregulates the expression of IFNγ, IL-8, IL-12, and IL-1β in *P. salmonis*-infected CHSE-214 cells. The elevated levels of these cytokines support bacterial survival within host cells by influencing the hepcidin/ferroportin axis, which plays an important role in regulating the availability of iron, a vital resource for the growth and survival of *P. salmonis* [38]. In the hosts, the inducible nitric oxide synthase (iNOS) and the subsequent nitric oxide (NO) release represents an important mediator of inflammatory events in many diseases [39]. However, the precise mechanism by which NO exercises its antibacterial activity is unknown and represents a significant gap in our understanding of why iNOS is considered to be in the first line of defence against bacterial infections given its proven bactericidal action [40]. Organic acids or their derivates (e.g., butyric acid) upregulate iNOS expression [19] potentially leading to an increased innate defence against pathogens such as *Renibacterium salmoninarum*, a pathogen known for causing kidney infections in trout [41]. The mixture of organic acids (Aq) used in our study also significantly increased the expression of iNOS and NO production in *P. salmonis*-infected CHSE-214 cells leading to a significant decrease in bacterial infection. Moreover, we report that the antimicrobial mixture (Aq) was able to increase the expression of cathelicidin (Cath2) and hepcidin (Hepcidin 1), antimicrobial peptides that are part of the innate immune function in *P. salmonis*- or *A. salmonicida*-infected hosts [42,43]. Under normal physiological conditions, NO plays an important anti-inflammatory effect and has a key role in the pathogenesis of inflammation [44]. Nitric oxide is indeed an important mediator and immunomodulator, an effect also extended to the expression of IFN-γ [45]. In our study, the increased NO release in the presence of Aq resulted in a significant inhibition of IFN-γ expression, emphasizing its anti-inflammatory effect. Nitric oxide also inhibits IL-12 synthesis in activated macrophages [46], an effect also observed in our study when infected CHSE-214 cells were exposed to 0.5% Aq. There is also historical evidence of NO-induced IL-8 production, indicating its role in mediating cytokine expression [47]. In our study, the Aq induced iNOS stimulation and the increased NO synthesis was not responsible for an increase in IL-8 expression, but, on the contrary, the production of this cytokine was significantly reduced. These results suggest that further mechanisms are engaged in the Aq-mediated reduction in IL-8 expression in our experimental design. Moreover, in the context of the decreased *P. salmonis* infection of CHSE-214 cells, in the presence of Aq, a lower IL-8 expression might be beneficial, as it has been suggested that IL-8 is a key chemokine in the control of bacterial translocation [48].

## 5. Conclusions

**Figure 7:**
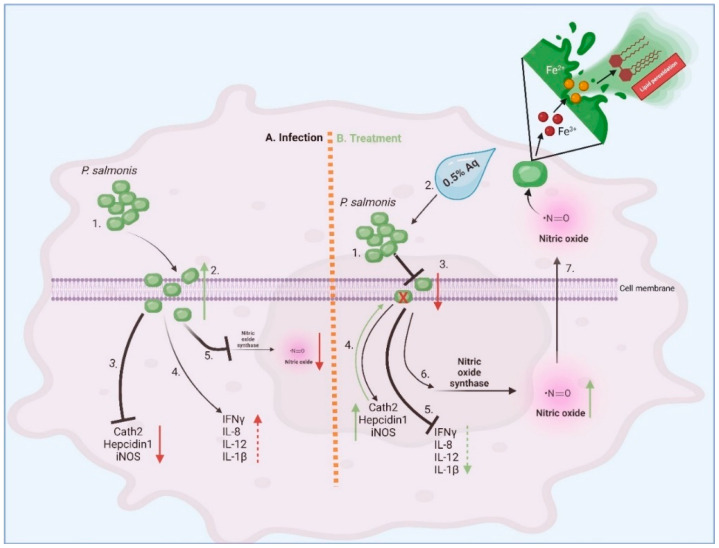
The antioxidant role of Aq in preventing *P. salmonis* infection of CHSE-214 epithelial cells. (**A**) Infection and untreated pathway; (**B**) infected and treated pathway.

Bacterial-induced, host-secreted reactive oxygen species were found to be responsible for imposing posttranslational modifications and phenotypic changes in cocultured bacteria with negative consequences on their virulence abilities [49]. Herein, we show that a blend of organic acids can stimulate the CHSE-214 epithelial cells to endogenously secrete nitric oxide, produce antimicrobial peptides, and reduce the pro-inflammatory upon *P. salmonis* infection (Figure 7). As reported in this study, the low levels of invasion could also be a result of NO-induced bacterial lipid peroxidation during coculture. All these biological events, mediated by the organic acid blend can potentially be considered an efficient first step in designing future interventions to prevent Salmonid Rickettsial Septicaemia and reduce the significant financial losses inflicted on farmers.

## Figures and Tables

**Table 1:** PCR primers.

Gene	Primer	Reference
Cath2	F: atgggaaacgaatgatgtgc R: cggtcagtgttgagggtatt	[19]
Hepcidin 1	F: gcttctgctgcaaattctgagg R: gtacaagattgaggttgtgcag	
iNOS	F: aacgagagccaacaggtgtc R: ggtgcagcatgtctttgaga	
IFNγ	F—ttcaggagacccagaaacactac R—taatgaactcggacagagccttc	AY795563.1
IL-8	F—gcaacagcggtcaggagatt R—tggaatgattccccttcttca	HM162835.1
IL-12	F—ctgaatgaggtggactggtatg R—atcgtcctgttcctccg	XM_014205516.1
IL-1β	F—caagctgcctcagggtct R—cggcaccctttaacctctcc	NM_001123582.1
16S rRNA	F—agggagactgccggtgata R—actacgaggcgctttctca	[20]
GADPH	F—ttccacggcacagtcaag R—actcagcaccagcatcac	[16]

## Data Availability

All data supporting our findings are included in the manuscript.

## References

[1] Xue X., Caballero-Solares A., Hall J.R., Umasuthan N., Kumar S., Jakob E., Skugor S., Hawes C., Santander J., Taylor R.G. (2021). Transcriptome Profiling of Atlantic Salmon (*Salmo salar*) Parr With Higher and Lower Pathogen Loads Following *Piscirickettsia salmonis* Infection. Front. Immunol..

[2] Caruffo M., Mandakovic D., Mejías M., Chávez-Báez I., Salgado P., Ortiz D., Montt L., Pérez-Valenzuela J., Vera-Tamargo F., Yánez J.M. (2020). Pharmacological iron-chelation as an assisted nutritional immunity strategy against *Piscirickettsia salmonis* infection. Vet. Res..

[3] Rozas-Serri M. (2022). Why Does *Piscirickettsia salmonis* Break the Immunological Paradigm in Farmed Salmon? Biological Context to Understand the Relative Control of Piscirickettsiosis. Front. Immunol..

[4] Yáñez J.M., Yoshida G.M., Parra Á., Correa K., Barría A., Bassini L.N., Christensen K.A., López M.E., Carvalheiro R., Lhorente J.P. (2019). Comparative Genomic Analysis of Three Salmonid Species Identifies Functional Candidate Genes Involved in Resistance to the Intracellular Bacterium *Piscirickettsia salmonis*. Front. Genet..

[5] Cortés H., Castillo-Ruiz M., Cañon-Jones H., Schlotterbeck T., San Martín R., Padilla L. (2023). In Vivo Efficacy of Purified Quillaja Saponin Extracts in Protecting against *Piscirickettsia salmonis* Infections in Atlantic Salmon (*Salmo salar)*. Animals.

[6] Laurin E., Gardner I.A., Peña A., Rozas-Serri M., Gayosa J., Neumann Heise J., Mardones F.O. (2020). Bayesian estimation of diagnostic sensitivity and specificity of a qPCR and a bacteriological culture method for *Piscirickettsia salmonis* in farmed Atlantic salmon (*Salmo salar* L.) in Chile. J. Fish. Dis..

[7] Godoy M., Coca Y., Suárez R., Montes de Oca M., Bledsoe J.W., Burbulis I., Caro D., Pontigo J.P., Maracaja-Coutinho V., Arias-Carrasco R. (2024). *Salmo salar* Skin and Gill Microbiome during *Piscirickettsia salmonis* Infection. Animals.

[8] Avendaño-Herrera R. (2021). Salmon aquaculture, *Piscirickettsia salmonis* virulence, and one health: Dealing with harmful synergies between heavy antimicrobial use and piscine and human health comment on Cabello and Godfrey (2019). Aquaculture.

[9] Santibañez N., Vega M., Pérez T., Yáñez A., González-Stegmaier R., Figueroa J., Enríquez R., Oliver C., Romero A. (2020). Biofilm Produced In Vitro by *Piscirickettsia salmonis* Generates Differential Cytotoxicity Levels and Expression Patterns of Immune Genes in the Atlantic Salmon Cell Line SHK-1. Microorganisms.

[10] Ghiselli F., Giovagnoni G., Felici M., Tugnoli B., Piva A., Grilli E. (2022). A mixture of organic acids and thymol protects primary chicken intestinal epithelial cells from Clostridium perfringens infection in vitro. Poult. Sci..

[11] Welsby I., Detienne S., N’Kuli F., Thomas S., Wouters S., Bechtold V., De Wit D., Gineste R., Reinheckel T., Elouahabi A. (2017). Lysosome-Dependent Activation of Human Dendritic Cells by the Vaccine Adjuvant QS-21. Front. Immunol..

[12] Balta I., Marcu A., Linton M., Kelly C., Stef L., Pet I., Ward P., Pircalabioru G.G., Chifiriuc C., Gundogdu O. (2021). The in vitro and in vivo anti-virulent effect of organic acid mixtures against Eimeria tenella and Eimeria bovis. Sci. Rep..

[13] Balta I., McCleery D., David S.R.F., Pet E., Stef D., Iancu T., Pet I., Stef L., Corcionivoschi N. (2024). The mechanistic role of natural antimicrobials in preventing Staphylococcus aureus invasion of MAC-T cells using an in vitro mastitis model. Ir. Vet. J..

[14] Pinkerton L., Linton M., Kelly C., Ward P., Gradisteanu Pircalabioru G., Pet I. (2019). Attenuation of vibrio parahaemolyticus virulence factors by a mixture of natural antimicrobials. Microorganisms.

[15] Cortés M.P., Mendoza S.N., Travisany D., Gaete A., Siegel A., Cambiazo V., Maass A. (2017). Analysis of *Piscirickettsia salmonis* Metabolism Using Genome-Scale Reconstruction, Modeling, and Testing. Front. Microbiol..

[16] Balta I., Marcu A., Linton M., Kelly C., Gundogdu O., Stef L., Pet I., Ward P., Deshaies M., Callaway T. (2021). Mixtures of natural antimicrobials can reduce Campylobacter jejuni, Salmonella enterica and Clostridium perfringens infections and cellular inflammatory response in MDCK cells. Gut Pathog..

[17] Henriquez M., Gonzalez E., Marshall S.H., Henriquez V., Gomez F.A., Martinez I., Altamirano C. (2013). A novel liquid medium for the efficient growth of the salmonid pathogen *Piscirickettsia salmonis* and optimization of culture conditions. PLoS ONE.

[18] Perez-Stuardo D., Morales-Reyes J., Tapia S., Ahumada D.E., Espinoza A., Soto-Herrera V., Brianson B., Ibaceta V., Sandino A.M., Spencer E. (2019). Non-lysosomal Activation in Macrophages of Atlantic Salmon (*Salmo salar*) after Infection with *Piscirickettsia salmonis*. Front. Immunol..

[19] Estevez R.A., Mostazo M.G.C., Rodriguez E., Espinoza J.C., Kuznar J., Jonsson Z.O., Guethmundsson G.H., Maier V.H. (2018). Inducers of salmon innate immunity: An in vitro and in vivo approach. Fish Shellfish Immunol..

[20] Karatas S., Mikalsen J., Steinum T.M., Taksdal T., Bordevik M., Colquhoun D.J. (2008). Real time PCR detection of *Piscirickettsia salmonis* from formalin-fixed paraffin-embedded tissues. J. Fish. Dis..

[21] Mangmool S., Limpichai C., Han K.K., Reutrakul V., Anantachoke N. (2022). Anti-Inflammatory Effects of Mitrephora sirikitiae Leaf Extract and Isolated Lignans in RAW 264.7 Cells. Molecules.

[22] Kolodziejski P.J., Koo J.S., Eissa N.T. (2004). Regulation of inducible nitric oxide synthase by rapid cellular turnover and cotranslational down-regulation by dimerization inhibitors. Proc. Natl. Acad. Sci. USA.

[23] Maier V.H., Schmitt C.N., Gudmundsdottir S., Gudmundsson G.H. (2008). Bacterial DNA indicated as an important inducer of fish cathelicidins. Mol. Immunol..

[24] Sheng H., Nakamura K., Kanno T., Sasaki K., Niwano Y. (2015). Bactericidal Effect of Photolysis of H_2_O_2_ in Combination with Sonolysis of Water via Hydroxyl Radical Generation. PLoS ONE.

[25] Levipan H.A., Irgang R., Opazo L.F., Araya-León H., Avendaño-Herrera R. (2022). Collective behavior and virulence arsenal of the fish pathogen *Piscirickettsia salmonis* in the biofilm realm. Front. Cell. Infect. Microbiol..

[26] Santibáñez N., Vega M., Pérez T., Enriquez R., Escalona C.E., Oliver C., Romero A. (2024). In vitro effects of phytogenic feed additive on *Piscirickettsia salmonis* growth and biofilm formation. J. Fish. Dis..

[27] Levipan H.A., Irgang R., Yáñez A., Avendaño-Herrera R. (2020). Improved understanding of biofilm development by *Piscirickettsia salmonis* reveals potential risks for the persistence and dissemination of piscirickettsiosis. Sci. Rep..

[28] Schairer D.O., Chouake J.S., Nosanchuk J.D., Friedman A.J. (2012). The potential of nitric oxide releasing therapies as antimicrobial agents. Virulence.

[29] Butucel E., Balta I., McCleery D., Marcu A., Stef D., Pet I., Callaway T., Stef L., Corcionivoschi N. (2022). The Prebiotic Effect of an Organic Acid Mixture on *Faecalibacterium prausnitzii* Metabolism and Its Anti-Pathogenic Role against *Vibrio parahaemolyticus* in Shrimp. Biology.

[30] Korhonen R., Korpela R., Saxelin M., Mäki M., Kankaanranta H., Moilanen E. (2001). Induction of nitric oxide synthesis by probiotic Lactobacillus rhamnosus GG in J774 macrophages and human T84 intestinal epithelial cells. Inflammation.

[31] Bunduruș I.A., Balta I., Butucel E., Callaway T., Popescu C.A., Iancu T., Pet I., Stef L., Corcionivoschi N. (2023). Natural Antimicrobials Block the Host NF-κB Pathway and Reduce Enterocytozoon hepatopenaei Infection Both In Vitro and In Vivo. Pharmaceutics.

[32] Jang B.C., Paik J.H., Kim S.P., Bae J.H., Mun K.C., Song D.K., Cho C.H., Shin D.H., Kwon T.K., Park J.W. (2004). Catalase induces the expression of inducible nitric oxide synthase through activation of NF-kappaB and PI3K signaling pathway in Raw 264.7 cells. Biochem. Pharmacol..

[33] Lee J.A., Song H.Y., Ju S.M., Lee S.J., Kwon H.-J., Eum W.S., Jang S.H., Choi S.Y., Park J. (2009). Differential regulation of inducible nitric oxide synthase and cyclooxygenase-2 expression by superoxide dismutase in lipopolysaccharide stimulated RAW 264.7 cells. Exp. Mol. Med..

[34] Hall J.R., Rouillard K.R., Suchyta D.J., Brown M.D., Ahonen M.J.R., Schoenfisc M.H. (2020). Mode of nitric oxide delivery affects antibacterial action. ACS Biomater. Sci. Eng..

[35] Jones M.L., Ganopolsky J.G., Labbe A., Wahl C., Prakash S. (2010). Antimicrobial properties of nitric oxide and its application in antimicrobial formulations and medical devices. Appl. Microbiol. Biotechnol..

[36] Cowley S.C., Myltseva S.V., Nano F.E. (1996). Phase variation in Francisella tularensis affecting intracellular growth, lipopolysaccharide antigenicity and nitric oxide production. Mol. Microbiol..

[37] Katsube T., Tsuji H., Onoda M. (2007). Nitric oxide attenuates hydrogen peroxide-induced barrier disruption and protein tyrosine phosphorylation in monolayers of intestinal epithelial cell. Biochim. Biophys. Acta.

[38] Oliver C., Coronado J.L., Martínez D., Kashulin-Bekkelund A., Lagos L.X., Ciani E., Sanhueza-Oyarzún C., Mancilla-Nova A., Enríquez R., Winther-Larsen H.C. (2023). Outer membrane vesicles from *Piscirickettsia salmonis* induce the expression of inflammatory genes and production of IgM in Atlantic salmon *Salmo salar*. Fish Shellfish Immunol..

[39] Cinelli M.A., Do H.T., Miley G.P., Silverman R.B. (2020). Inducible nitric oxide synthase: Regulation, structure, and inhibition. Med. Res. Rev..

[40] Chakravortty D., Hensel M. (2003). Inducible nitric oxide synthase and control of intracellular bacterial pathogens. Microbes Infect..

[41] Campos-Perez J.J., Ward M., Grabowski P.S., Ellis A.E., Secombes C.J. (2000). The gills are an important site of iNOS expression in rainbow trout *Oncorhynchus mykiss* after challenge with the gram-positive pathogen Renibacterium salmoninarum. Immunology.

[42] Alvarez C.A., Guzman F., Cardenas C., Marshall S.H., Mercado L. (2014). Antimicrobial activity of trout hepcidin. Fish Shellfish Immunol..

[43] Douglas S.E., Gallant J.W., Liebscher R.S., Dacanay A., Tsoi S.C. (2003). Identification and expression analysis of hepcidin-like antimicrobial peptides in bony fish. Dev. Comp. Immunol..

[44] Sharma J.N., Al-Omran A., Parvathy S.S. (2007). Role of nitric oxide in inflammatory diseases. Inflammopharmacology.

[45] Tripathi P., Tripathi P., Kashyap L., Singh V. (2007). The role of nitric oxide in inflammatory reactions. FEMS Immunol. Med. Microbiol..

[46] Huang F.P., Niedbala W., Wei X.Q., Xu D., Feng G.J., Robinson J.H., Lam C., Liew F.Y. (1998). Nitric oxide regulates Th1 cell development through the inhibition of IL-12 synthesis by macrophages. Eur. J. Immunol..

[47] Villarete L.H., Remick D.G. (1997). Nitric oxide regulation of interleukin-8 gene expression. Shock.

[48] Sansonetti P.J., Arondel J., Huerre M., Harada A., Matsushima K. (1999). Interleukin-8 controls bacterial transepithelial translocation at the cost of epithelial destruction in experimental shigellosis. Infect. Immun..

[49] Corcionivoschi N., Alvarez L.A., Sharp T.H., Strengert M., Alemka A., Mantell J. (2012). Mucosal reactive oxygen species decrease virulence by disrupting Campylobacter jejuni phosphotyrosine signaling. Cell Host Microbe.

